# Migrants’ primary care utilisation before and during the COVID-19 pandemic in England: An interrupted time series analysis

**DOI:** 10.1016/j.lanepe.2022.100455

**Published:** 2022-06-30

**Authors:** Claire X. Zhang, Yamina Boukari, Neha Pathak, Rohini Mathur, Srinivasa Vittal Katikireddi, Parth Patel, Ines Campos-Matos, Dan Lewer, Vincent Nguyen, Greg C.G. Hugenholtz, Rachel Burns, Amy Mulick, Alasdair Henderson, Robert W. Aldridge

**Affiliations:** a Institute of Health Informatics, University College London, 222 Euston Rd, London NW1 2DA, United Kingdom; b Office for Health Improvement and Disparities, Department of Health and Social Care, 39 Victoria Street, London SW1H 0EU, United Kingdom; c National Perinatal Epidemiology Unit, Nuffield Department of Population Health, University of Oxford, Richard Doll Building, Old Road Campus, Headington, Oxford OX3 7LF, United Kingdom; d Guy's & St Thomas's NHS Foundation Trust, London SE1 9RT, United Kingdom; e Department of Non-communicable Disease Epidemiology, London School of Hygiene & Tropical Medicine, Keppel Street, London WC1E 7HT, United Kingdom; f MRC/CSO Social & Public Health Sciences Unit, University of Glasgow, Berkeley Square, 99 Berkeley Street, Glasgow G3 7HR, United Kingdom; g UK Health Security Agency, Wellington House, 133–155, Waterloo Road, London SE1 8UG, United Kingdom; h Institute of Epidemiology and Health Care, University College London, 1-19 Torrington Place, London WC1E 7HB, United Kingdom

**Keywords:** Migrant, Primary care, Consultation, General practice, COVID-19, Healthcare utilisation, Healthcare access

## Abstract

**Background:**

How international migrants access and use primary care in England is poorly understood. We aimed to compare primary care consultation rates between international migrants and non-migrants in England before and during the COVID–19 pandemic (2015–2020).

**Methods:**

Using data from the Clinical Practice Research Datalink (CPRD) GOLD, we identified migrants using country-of-birth, visa-status or other codes indicating international migration. We linked CPRD to Office for National Statistics deprivation data and ran a controlled interrupted time series (ITS) using negative binomial regression to compare rates before and during the pandemic.

**Findings:**

In 262,644 individuals, pre-pandemic consultation rates per person-year were 4.35 (4.34–4.36) for migrants and 4.60 (4.59–4.60) for non-migrants (RR:0.94 [0.92–0.96]). Between 29 March and 26 December 2020, rates reduced to 3.54 (3.52–3.57) for migrants and 4.2 (4.17–4.23) for non-migrants (RR:0.84 [0.8–0.88]). The first year of the pandemic was associated with a widening of the gap in consultation rates between migrants and non-migrants to 0.89 (95% CI 0.84–0.94) times the ratio before the pandemic. This widening in ratios was greater for children, individuals whose first language was not English, and individuals of White British, White non-British and Black/African/Caribbean/Black British ethnicities. It was also greater in the case of telephone consultations, particularly in London.

**Interpretation:**

Migrants were less likely to use primary care than non-migrants before the pandemic and the first year of the pandemic exacerbated this difference. As GP practices retain remote and hybrid models of service delivery, they must improve services and ensure primary care is accessible and responsive to migrants’ healthcare needs.

**Funding:**

This study was funded by the Medical Research Council (MC_PC 19070 and MR/V028375/1) and a Wellcome Clinical Research Career Development Fellowship (206602).

**Research in context**

**Evidence before this study**

How international migrants access and use primary care services in England, including the effect of the COVID-19 pandemic on access and usage, is poorly understood. We conducted literature searches in MEDLINE and Embase for articles published from 2002 to 2 February 2022 on migrants’ primary care access and utilisation in the UK, including during the pandemic and studies using data from the Clinical Practice Research Datalink (CPRD). We found 37 studies that examined migrants’ primary care access and utilisation in the UK, with 34 of these conducted before the pandemic. Of these 34, the majority had limited geographical coverage with small sample sizes, employed a qualitative design, included only individuals with specific conditions (e.g. HIV), or used survey methods to measure quantitative outcomes. There were only three large-scale studies that described migrants’ primary care access or utilisation. Two studies that used linked data for individuals from countries with a high incidence of TB found low levels of primary care registration in this migrant group. The third, a population-wide study, linked prescription data with data from the 2011 Census in Northern Ireland, reported reduced dispensing of psychotropic medication in most migrants compared to non-migrants.

There is little evidence on the impact of the pandemic on migrants’ primary care utilisation. Of the three studies that examined this, two employed qualitative designs, and one mixed-methods study analysed data from vulnerable migrants who accessed volunteer-led clinics. All three studies suggest that the COVID-19 pandemic may have exacerbated existing inequalities. However, no large-scale studies have used electronic health records to compare primary care utilisation in migrants in England before and during the pandemic.

**Added value of this study**

This study explored migrants’ primary care utilisation in England both prior to and during the COVID-19 pandemic in a large cohort of over 130,000 migrants. We found that migrants were less likely than non-migrants to use primary care services before the pandemic and that the gap between migrants and non-migrants increased during the first year of the pandemic. This widening of the gap in consultation rates was greater in children, certain ethnic groups, and migrants whose first language was not English.

**Implications of all the available evidence**

Building on existing qualitative evidence, this study shows that migrants’ use of primary care in England was disproportionately impacted by the COVID-19 pandemic. As models of primary care service delivery continue to change in response to the pandemic, policy-makers, commissioners, and service planners must ensure that primary care is accessible and adequately resourced to understand and meet the diverse needs of their local migrant and ethnic communities across different age groups, especially individuals whose first language is not English.

Alt-text: Unlabelled box

## Introduction

Effective primary care is linked to better health outcomes in the general population.^1^ In the United Kingdom (UK), access to primary care is free of charge for all.^2^ However, barriers to general practice (GP) registration and low registration rates are long-standing issues among international migrants, particularly those who are socially excluded.^3^, ^4^, ^5^, ^6^, ^7^ For migrants who do register, there are barriers to accessing care, including insufficient translation support, discrimination, and transportation costs.^8^, ^9^, ^10^ However, differences in primary care utilisation between migrants and the UK-born population are poorly understood and have relied on self-reported surveys with limited sample sizes and mixed results.^11^^,^^12^ Examining these differences is of particular importance for service planning given the UK is home to the fifth largest number of international migrants in the world.^13^

Substantial reductions in primary care consultations were observed across the UK during the first wave of the COVID-19 pandemic,^14^^,^^15^ with people from minority ethnic groups reporting greater healthcare disruption than individuals from White ethnic groups.^16^ However, the difference in primary care utilisation between migrants and non-migrants in England and how the pandemic has affected this, including the important interplay between migration and ethnicity within increasingly diverse communities, has not been studied. This is needed to help identify inequalities, and to inform service provision and policy.^17^ Shifts from in-person to remote consultations may make primary care access even more challenging for people at risk of digital exclusion, including some migrants,^18^ although no association was found between deprivation status and the likelihood of accessing remote consultations.^19^

Recent validation of an electronic health record (EHR) code list to identify a cohort of migrants largely representative of the broader migrant population^20^ presents a unique opportunity to quantify differences in primary care usage between migrants and non-migrants in England before and during the COVID-19 pandemic.

This study aimed to compare National Health Service (NHS) primary care consultation rates between migrants and non-migrants from 2015 to 2020 in England, specifically:

1. Did consultation rates differ between migrants and non-migrants before the pandemic?
2. Did this change during the pandemic?
3. Did differences between migrants and non-migrants vary across ethnic groups?

## Methods

### Study overview and data management

**Figure 1:**
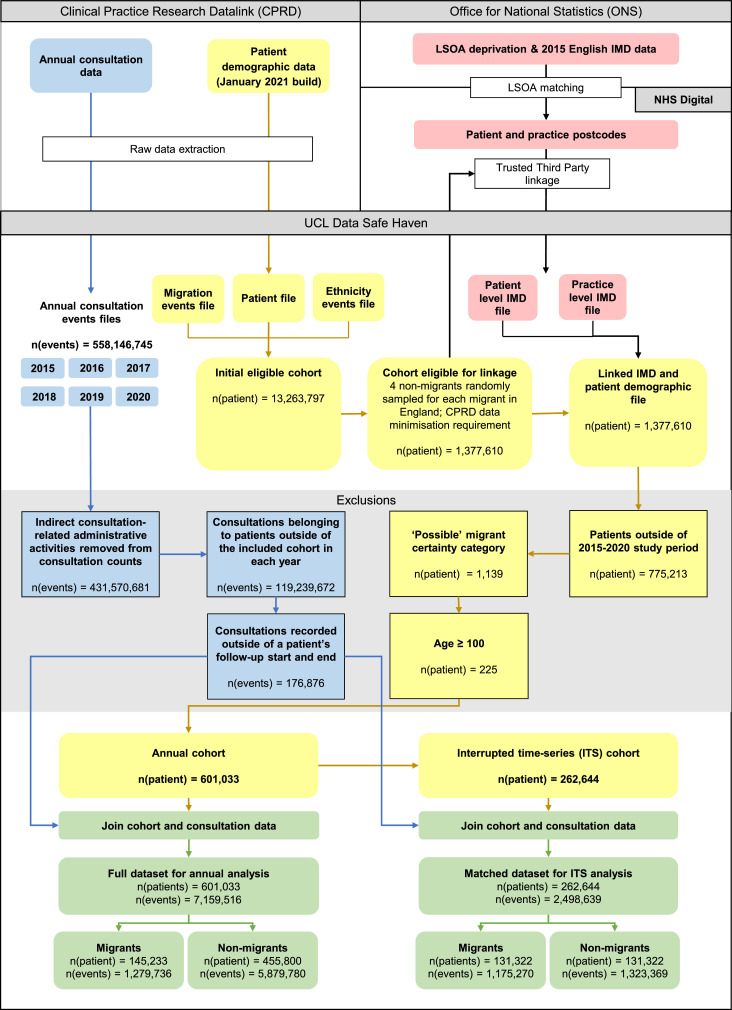
Data flow diagram with patient and consultation exclusions.

Of over 900 GP practices in the UK contributing to Clinical Practice Research Datalink (CPRD) GOLD, 413 were in England and had linked Office for National Statistics (ONS) 2015 Index of Multiple Deprivation (IMD) data. Data flows are shown in Figure 1. Pseudo-anonymised data were stored, cleaned, and analysed using R (versions 3.6.2 and 4.0.3) in the University College London Data Safe Haven during 2021. All code for data cleaning and analysis is freely available https://doi.org/10.5281/zenodo.6345286.

This study was approved by the UK Medicines and Healthcare products Regulatory Agency Independent Scientific Advisory Committee (protocol 19_062R, approval on 29 April 2019) and it was carried out as part of the CALIBER programme under Section 251 (NHS Social Care Act 2006), which has NHS research ethics approval (09/H0810/16).

### Exposure and outcomes

The exposure of interest was international migration to the UK, determined by a validated migration code list comprising migration-related ‘Read Version 2’ codes.^20^ The code list produces a binary indicator (migrants, non-migrants). It also disaggregates migrants into levels of certainty: ‘definite’ migrants with country-of-birth and/or visa-status codes, ‘probable’ migrants with main-language-other-than-English codes, and ‘possible’ migrants with non-UK-origin codes.

The outcomes were primary care consultation rates (per person-year) and rate ratios (RRs) comparing migrants to non-migrants. We derived consultation counts using 28 out of 62 consultation types in CPRD indicating direct consultations (as opposed to indirect administrative activities) and further disaggregated them into face-to-face and telephone consultations, the latter of which may also include other types of virtual consultations (Table S1).

### Study cohort

The initial eligible cohort comprised individuals of all ages registered before January 2021, for any length of time, at a CPRD GOLD GP practice in the UK that was contributing ‘up-to-standard’ data (see Supplementary Box 2 for details) for any length of time in the January 2021 database build.^21^ We reduced this initial cohort to individuals registered at a GP practice in England who were eligible for all linkages pre-specified in the study protocol^22^ and we identified migrants by applying the migration code list. To comply with CPRD's data minimisation policy, we randomly sampled non-migrants from the reduced initial cohort at a ratio of 1:4 migrants to non-migrants and then linked this cohort to IMD data.

Follow-up commenced at the latest date of: a) an individual registering at a CPRD GOLD practice; b) their practice's first ‘up-to-standard’ date; c) 1 January 2015. Follow-up ended at the earliest date of: a) a patient's transfer out of a CPRD GOLD practice; b) their date of death; c) the last data collection date for the practice; d) 26 December 2020 (end date of available data).

We made exclusions at the patient and consultation level (Figure 1 and Supplementary Box 2). To prioritise specificity, we excluded ‘possible’ migrants due to uncertainty around their migration status.

### Statistical analysis: before the pandemic

We compared pre-pandemic annual consultation rates between migrants and non-migrants using RRs derived from unadjusted and adjusted negative binomial models to account for overdispersion in the data. Covariates were individual's time-varying age (i.e., their age in each study year represented by 5-year age categories), sex, study year, and practice region; the offset was the log person-years of follow-up. As socioeconomic status is commonly recognised as a mediator of the relationship between ethnicity and healthcare,^23^ we considered it a potentially important mediator of the effect of migration on consultation rates. We, therefore, ran multivariable models with and without adjusting for index of multiple deprivation (IMD; prioritising patient-postcode-level IMD for individuals with linked IMD data over practice-postcode-level IMD). We stratified models by larger age groups (0–15, 16–24, 25–34, 35–49, 50–64, and 65 and over) to account for differences in representativeness of the migration code list across different age groups.^20^ Due to interest from London policymakers who were part of the steering group for the study grant, we conducted a secondary analysis of individuals in London only.

### Statistical analysis: before versus during the pandemic

We explored any changes in the gap between migrants’ and non-migrants’ consultation rates before versus during the pandemic via an interrupted time series (ITS) analysis using a step-change model adapted from Mansfield et al.^14^ (see Supplementary Box 3). We compared the pandemic period, defined as the time following introduction of national restrictions (29 March 2020 to 26 December 2020), with the pre-pandemic period (4 January 2015 to 7 March 2020). We added an interaction term between the pandemic period and migration status, which is interpreted as the change in the ratio of consultation rates between migrants and non-migrants during the pandemic compared to the consultation rate ratio between migrants and non-migrants before the pandemic (i.e., the multiplicative effect of migration). We did not include a recovery slope term as our focus was on the average effect of migration on consultation rates during the initial months of the pandemic. We removed data for 8–28 March 2020 to account for healthcare utilisation changes in anticipation of pandemic restrictions. We matched migrants and non-migrants in the ITS cohort on a 1:1 ratio by sex, practice region, IMD and age at study start. In secondary analyses, we limited the analysis to London, and also stratified by consultation type (face-to-face and telephone) given considerable changes to service delivery during the pandemic.

### Statistical analysis: effect modification by ethnicity

In the pre-pandemic analysis, we examined effect modification by ethnicity using a two-way interaction term between migration and ethnicity, based on the 2011 Census’ 18 categories grouped into 6 broader categories and derived using an ethnicity code list adapted from Pathak et al. (Table S2), with an ‘Unknown’ category for individuals with no ethnicity data.^20^ We calculated the relative excess risk due to interaction (RERI, additive)^24^ and multiplicative effects. We also calculated the effect of migration across each ethnicity strata, and compared migrants of each ethnicity to White British non-migrants.^25^ In the ITS analysis, we included a three-way interaction term between migration, ethnicity and pandemic to determine the effect of migration across each ethnicity strata (see Supplementary Box 3).

### Bias

We replaced the binary migrant status with the categorical migration certainty variable (‘definite’ and ‘probable’ migrants) to assess potential misclassification bias in the annual pre-pandemic and ITS analyses. To examine any potential bias introduced by migrants’ younger age at cohort entry, later cohort entry, shorter time between entering the CPRD GOLD database and entering the cohort, and shorter follow-up time, we conducted two pre-pandemic and one ITS sensitivity analyses. First, we matched migrants and non-migrants on a 1:1 ratio by practice region, and age and year at entering CPRD GOLD. Second, we replaced the age and year at entering CPRD GOLD matching variables with follow-up time. Finally, we conducted an ITS sensitivity analysis by matching on age and year at entering CPRD GOLD (in addition to sex, practice region, and IMD). To examine potential bias introduced by matching migrants to non-migrants by deprivation level and geographic region, we conducted two further ITS sensitivity analyses; firstly, by matching on age at study start, sex, and practice region; and secondly, matching on age at study start and sex only. We visually inspected RRs from the pre-pandemic annual analysis and ITS analysis for differences.

### Role of the funding source

The funder of this study had no role in the study design, data collection, analysis, interpretation, writing of this report, or in the decision to submit the paper for publication.

## Results

### Cohort characteristics

The pre-pandemic annual cohort comprised 601,033 individuals, of which 145,233 were migrants. Migrants were younger than non-migrants at cohort entry and exit, and their median time between entering CPRD GOLD and entering the study cohort was shorter (Table S3). These differences were generally attenuated in the matched annual-analysis cohorts (Tables S4 and S5).

The ITS cohort comprised 262,644 individuals, with half identified as migrants (Table 1). A greater proportion of migrants and non-migrants were located in London versus other regions. Fewer migrants were of White British ethnicity (3.2%) compared to non-migrants (44.6%). The proportion of White British non-migrants is lower than the national average,^26^ possibly due to unrecorded ethnicity for 31.7% of individuals. Migrants had a shorter time between CPRD GOLD entry and study entry compared with non-migrants (median of 0.4 and 3.8 years, respectively). However, matching migrants to non-migrants based on age and year of CPRD GOLD entry removed this difference (Table S6).

**Table 1:** Demographic characteristics of the ITS cohort (matched on age at study start, sex, practice region, and IMD).

Characteristic	Overall*N* = 262,644	Non-migrant*n* = 131,322 (50.0%)	Migrant*n* = 131,322 (50.0%)	Definite migrant *n* = 46,583 (35.5%)	Probable migrant *n* = 84,739 (64.5%)
*Follow up, person–years*					
Total	563,116	289,267	273,849	90,894	182,956
Mean (SD)	2.14 (1.89)	2.20 (1.92)	2.09 (1.86)	1.95 (1.85)	2.16 (1.85)
Median (IQR)	1.55 (2.89)	1.55 (2.95)	1.55 (2.80)	1.39 (2.78)	1.65 (2.78)
*Sex, n (%)*					
Male	126,108 (48.0%)	63,054 (48.0%)	63,054 (48.0%)	22,812 (49.0%)	40,242 (47.5%)
Female	136,536 (52.0%)	68,268 (52.0%)	68,268 (52.0%)	23,771 (51.0%)	44,497 (52.5%)
*Year of cohort entry, n (%)*					
2015	196,541 (74.8%)	106,327 (81.0%)	90,214 (68.7%)	36,601 (78.6%)	53,613 (63.3%)
2016	17,470 (6.7%)	6642 (5.1%)	10,828 (8.2%)	3326 (7.1%)	7502 (8.9%)
2017	16,001 (6.1%)	6752 (5.1%)	9249 (7.0%)	2396 (5.1%)	6853 (8.1%)
2018	14,520 (5.5%)	5256 (4.0%)	9264 (7.1%)	2112 (4.5%)	7152 (8.4%)
2019	16,368 (6.2%)	5171 (3.9%)	11,197 (8.5%)	2035 (4.4%)	9162 (10.8%)
2020	1744 (0.7%)	1174 (0.9%)	570 (0.4%)	113 (0.2%)	457 (0.5%)
*Age at cohort entry, years*					
Mean (SD)	33 (18)	33 (18)	33 (18)	35 (17)	32 (19)
Median (IQR)	32 (21)	32 (21)	32 (21)	33 (20)	32 (24)
*Age at cohort exit, years*					
Mean (SD)	35 (18)	35 (18)	35 (18)	36 (17)	34 (19)
Median (IQR)	34 (22)	34 (22)	34 (22)	35 (21)	34 (24)
*Time between CPRD GOLD entry and study cohort entry, years*					
Mean (SD)	4.3 (5.8)	6.2 (6.7)	2.4 (3.8)	2.5 (3.7)	2.4 (3.8)
Median (IQR)	1.8 (6.4)	3.8 (11.3)	0.4 (3.5)	0.4 (3.7)	0.4 (3.4)
*Ethnicity, n (%)*					
White British	62,762 (23.9%)	58,561 (44.6%)	4201 (3.2%)	1883 (4.0%)	2318 (2.7%)
White non–British	63,118 (24.0%)	12,036 (9.2%)	51,082 (38.9%)	13,177 (28.3%)	37,905 (44.7%)
Mixed/Multiple ethnic groups	6344 (2.4%)	2456 (1.9%)	3888 (3.0%)	1542 (3.3%)	2346 (2.8%)
Asian/Asian British	43,126 (16.4%)	7415 (5.6%)	35,711 (27.2%)	9858 (21.2%)	25,853 (30.5%)
Black/African/Caribbean/Black British	19,442 (7.4%)	7642 (5.8%)	11,800 (9.0%)	6030 (12.9%)	5770 (6.8%)
Other ethnic group	10,475 (4.0%)	1572 (1.2%)	8903 (6.8%)	2586 (5.6%)	6317 (7.5%)
Unknown	57,377 (21.8%)	41,640 (31.7%)	15,737 (12.0%)	11,507 (24.7%)	4230 (5.0%)
*Practice region, n (%)*^a^					
London	100,020 (38.1%)	50,010 (38.1%)	50,010 (38.1%)	22,003 (47.2%)	28,007 (33.1%)
North East	984 (0.4%)	492 (0.4%)	492 (0.4%)	77 (0.2%)	415 (0.5%)
North West	28,982 (11.0%)	14,491 (11.0%)	14,491 (11.0%)	4769 (10.2%)	9722 (11.5%)
Yorkshire & The Humber	612 (0.2%)	306 (0.2%)	306 (0.2%)	73 (0.2%)	233 (0.3%)
East Midlands	64 (0.0%)	32 (0.0%)	32 (0.0%)	10 (0.0%)	22 (0.0%)
West Midlands	20,080 (7.6%)	10,040 (7.6%)	10,040 (7.6%)	1333 (2.9%)	8707 (10.3%)
East of England	14,326 (5.5%)	7163 (5.5%)	7163 (5.5%)	957 (2.1%)	6206 (7.3%)
South West	16,306 (6.2%)	8153 (6.2%)	8153 (6.2%)	3692 (7.9%)	4461 (5.3%)
South Central	40,764 (15.5%)	20,382 (15.5%)	20,382 (15.5%)	10,232 (22.0%)	10,150 (12.0%)
South East Coast	40,506 (15.4%)	20,253 (15.4%)	20,253 (15.4%)	3437 (7.4%)	16,816 (19.8%)
*IMD, n (%)*					
IMD 1 (least deprived)	36,336 (13.8%)	18,168 (13.8%)	18,168 (13.8%)	7502 (16.1%)	10,666 (12.6%)
IMD 2	38,976 (14.8%)	19,488 (14.8%)	19,488 (14.8%)	6116 (13.1%)	13,372 (15.8%)
IMD 3	48,688 (18.5%)	24,344 (18.5%)	24,344 (18.5%)	7742 (16.6%)	16,602 (19.6%)
IMD 4	66,990 (25.5%)	33,495 (25.5%)	33,495 (25.5%)	11,548 (24.8%)	21,947 (25.9%)
IMD 5 (most deprived)	71,654 (27.3%)	35,827 (27.3%)	35,827 (27.3%)	13,675 (29.4%)	22,152 (26.1%)
*Patients in each study year, n (%)*					
2015	196,541 (27.5%)	106,327 (29.4%)	90,214 (25.6%)	36,601 (31.2%)	53,613 (22.8%)
2016	138,899 (19.5%)	73,028 (20.2%)	65,871 (18.7%)	24,212 (20.6%)	41,659 (17.7%)
2017	115,280 (16.1%)	59,524 (16.5%)	55,756 (15.8%)	16,921 (14.4%)	38,835 (16.5%)
2018	103,295 (14.5%)	49,249 (13.6%)	54,046 (15.3%)	16,314 (13.9%)	37,732 (16.0%)
2019	94,498 (13.2%)	43,561 (12.1%)	50,937 (14.5%)	13,417 (11.4%)	37,520 (16.0%)
2020	65,296 (9.1%)	29,697 (8.2%)	35,599 (10.1%)	9857 (8.4%)	25,742 (10.9%)

a See Supplementary Box 4 for a map of England and its regions.

### Before the pandemic

Migrants in the annual cohort attended fewer consultations than non-migrants; 4.31 (4.31–4.32) compared with 5.62 (5.62–5.62) consultations per person-year (Table S9). A similar, although less pronounced, trend was observed in the pre-pandemic period of the ITS analysis (4.35 [4.34–4.36] versus 4.6 [4.59–4.6] consultations per person–year; Table S10).

**Figure 2:**
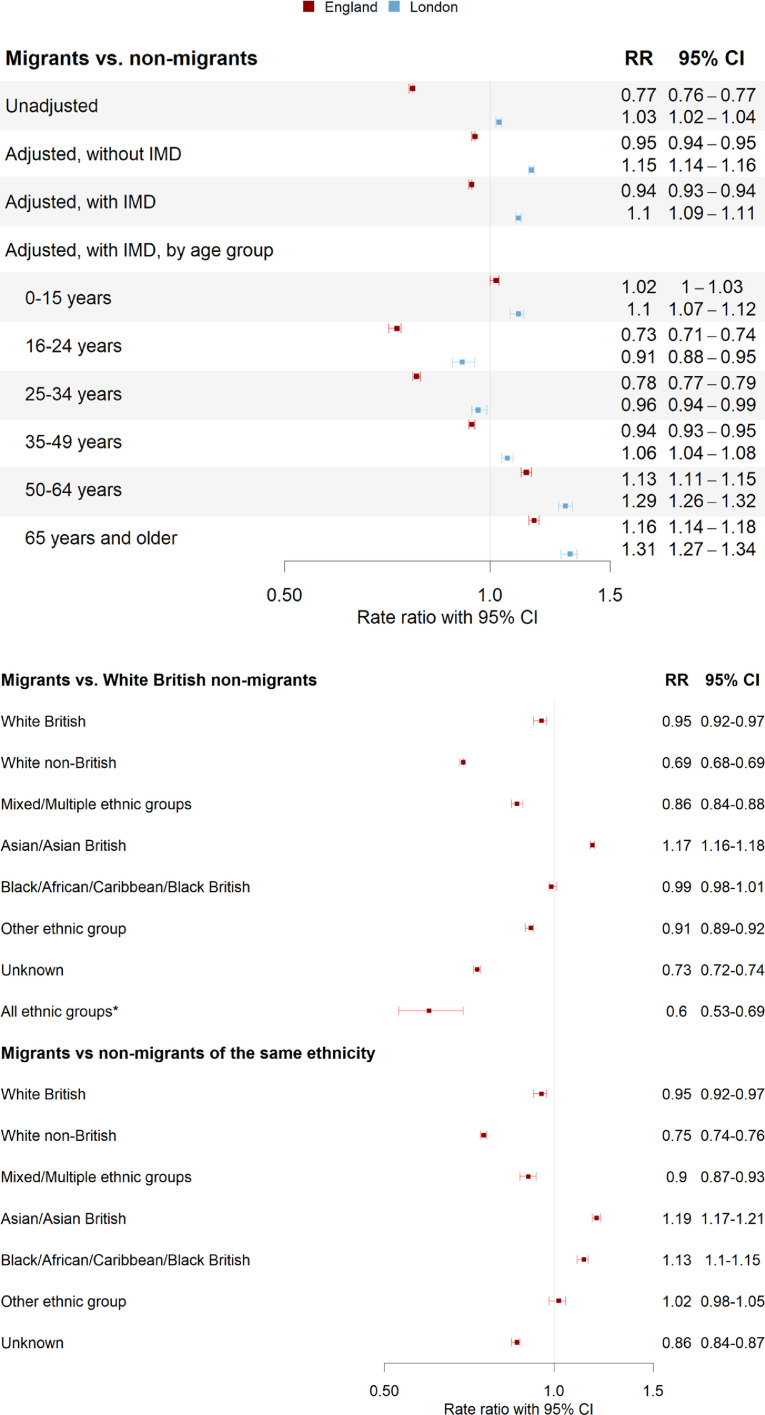
Forest plots of migrant versus non–migrant consultation rate ratios before the pandemic (2015–2019), including by age group (A) and ethnicity (B). *All represents migrants of all ethnic groups compared to White British non-migrants.

Migrants had a 6% lower rate of consultations than non-migrants after multivariable adjustment (Figure 2A and Table S11, RR:0.94, 95%CI:0.93–0.94). A similar RR was obtained when IMD was removed from the model (RR:0.95, 95%CI:0.94–0.95). Consultation rates in migrants were slightly higher than non-migrants for individuals aged 0–15 years, 50–64 years, and 65 years and above, while migrants aged 16–24 years, 25–34 years, and 35–49 years had lower consultation rates than non-migrants (Figure 2A).

In London, migrants had higher rates of consultation than non-migrants after multivariable adjustment (Figure 2A; RR:1.10, 95%CI:1.09–1.11). Consultation rates were only lower among migrants aged 16–24 years and 25–34 years. However, the differences in consultation rates between migrants and non-migrants in these age groups were not as great as those seen in the same age groups in England.

### Before versus during the pandemic

Crude face-to-face consultation rates reduced during the first eight months of the pandemic from 4.12 (4.11–4.13) to 3.02 (3–3.05) and 4.35 (4.34–4.36) to 3.52 (3.49–3.55) consultations per person-year for migrants and non-migrants, respectively (Table S10). Telephone consultations increased during the pandemic in both groups (from 0.23 [0.23–0.23] to 0.52 [0.51–0.53] in migrants and 0.25 [0.25–0.25] to 0.68 [0.67–0.69] in non-migrants).

**Figure 3:**
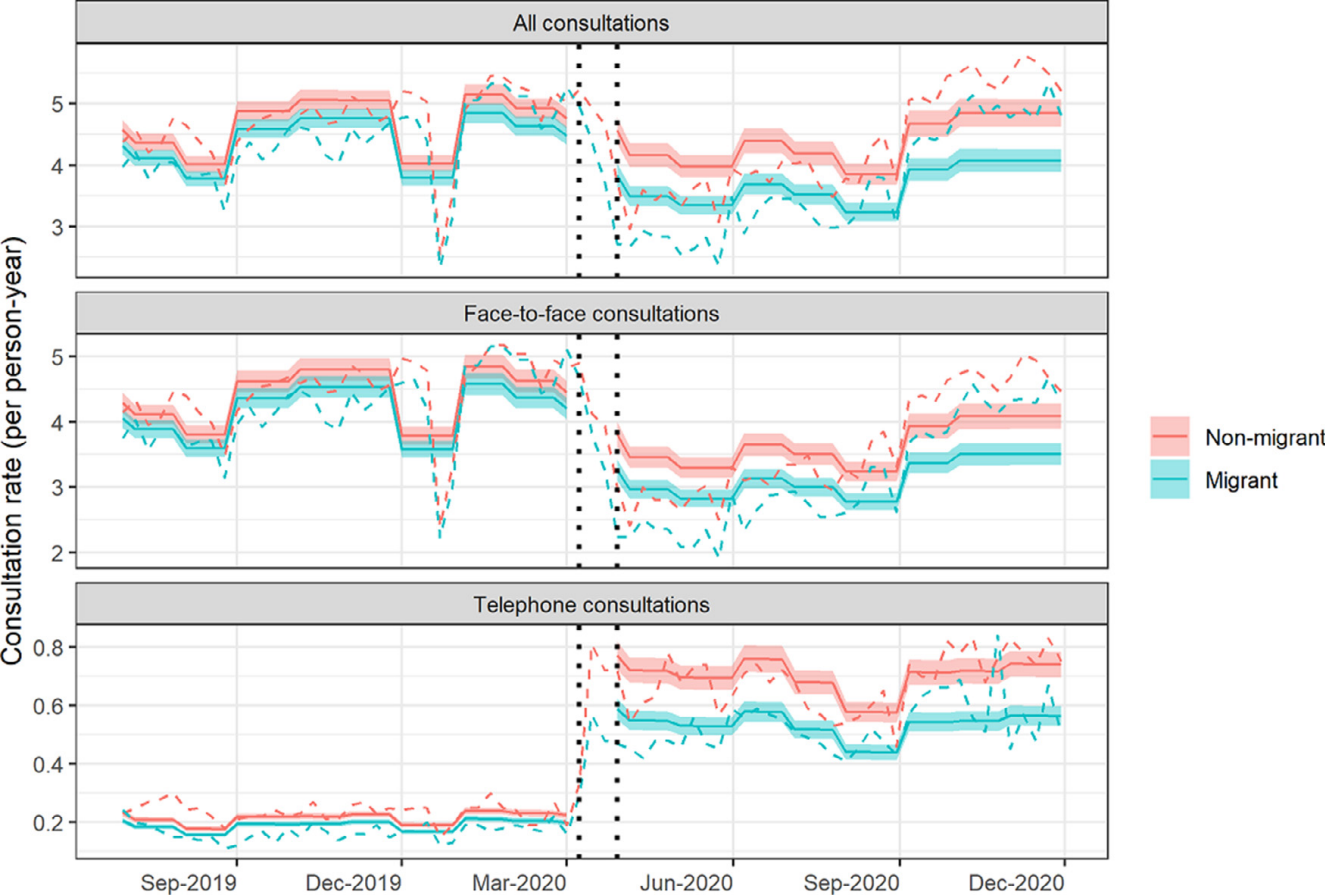
Weekly consultation rates by migration status in England: predicted rates from interrupted time-series analysis (solid line) and actual observed rates (dashed line), truncated view July 2019–November 2020.

During the pandemic, migrants had lower rates of face-to-face (RR:0.86, 95%CI:0.81–0.9) and telephone consultations (RR:0.76, 95%CI:0.71–0.81) compared to non-migrants (Figure 3, Table 2). This resulted in a 9% reduction (RR:0.91, 95%CI:0.86–0.96) in the ratio of face-to-face consultation rates observed between migrants and non-migrants pre-pandemic, and a 14% reduction for telephone consultations (RR:0.86, 95%CI:0.80–0.92).

**Table 2:** Consultation rate ratios from interrupted time-series analysis (5 January 2015 to 26 December 2020) for England.

Variable	Interpretation	RR (95%CI)		
		All	Face-to-face	Telephone
Pandemic	Comparing non-migrants’ consultation rates during the pandemic to non-migrants’ consultation rates pre-pandemic	0.96 (0.92–1)	0.85 (0.82–0.89)	3.45 (3.26–3.65)
Migration status	Comparing migrants’ consultation rates pre-pandemic to non-migrants consultations pre-pandemic	0.94 (0.92–0.96)	0.94 (0.93–0.96)	0.89 (0.86–0.91)
Migration status + interaction term (between migrant status and pandemic)	Comparing migrants’ consultation rates during the pandemic to non-migrants’ consultation rates during the pandemic	0.84 (0.8–0.88)	0.86 (0.81–0.9)	0.76 (0.71–0.81)
Interaction term (between migrant status and pandemic)	Comparing the ratio of consultation rates between migrants and non-migrants pre-pandemic to the ratio of consultation rates between migrants and non-migrants during the pandemic (the additional effect of the pandemic on the ratio between migrants and non-migrants i.e. the multiplicative effect)	0.89 (0.84–0.94)	0.91 (0.86–0.96)	0.86 (0.8–0.92)

**Figure 4:**
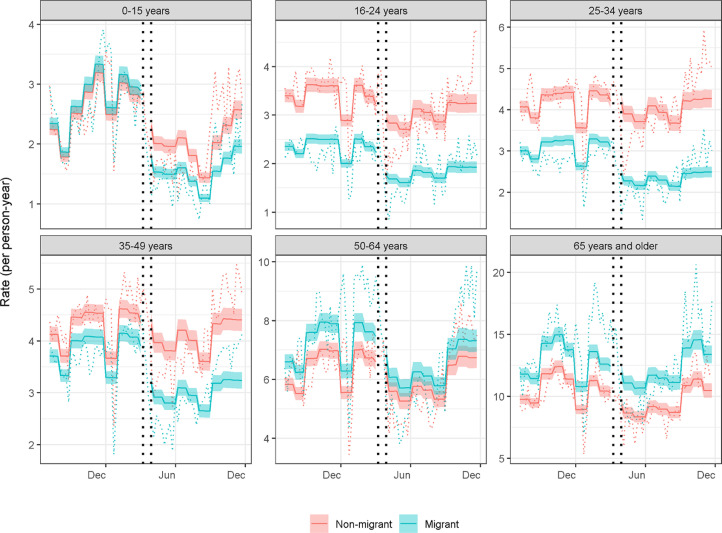
Weekly consultation rates by migration status and age group in England: predicted rates from interrupted time-series analysis (solid line) and actual observed rates (dashed line), truncated view July 2019–November 2020.

This change was more pronounced in migrants aged 0–15 years, who attended more consultations before the pandemic (RR:1.05, 95%CI:1.02–1.07) and fewer during the pandemic (RR:0.76, 95%CI:0.71–0.82). It resulted in a 27% reduction in the pre-pandemic ratio of consultation rates between migrants and non-migrants (RR:0.73, 95%CI:0.68–0.79). Other age groups were also negatively affected to varying degrees (Figure 4 and Table S12).

In London, migrants had slightly higher rates both before and during the pandemic (Table S13). Migrants and non-migrants in London had similar rates of telephone consultations pre-pandemic (RR:0.99, 95%CI:0.95–1.03; Figure S1 and Table S13). However, during the pandemic migrants had substantially lower rates than non-migrants (RR:0.77, 95%CI:0.70–0.86), which equates to a further 22% reduction compared with the pre-pandemic ratio of consultation rates between migrants and non-migrants (RR:0.78, 95%CI:0.7–0.88).

### Effect modification by ethnicity

Pre-pandemic, White non-British migrants had the lowest consultation rates compared to non-migrants of the same ethnicity (Figure 2B Figure 2; RR:0.75, 95%CI:0.74–0.76), followed by migrants of Unknown, Mixed/Multiple, and White British ethnicities. Conversely, Asian/Asian British migrants (RR:1.19, 95%CI:1.17–1.21) and Black/African/Caribbean/Black British migrants (RR:1.13, 95%CI:1.1–1.15) had higher consultation rates than their non-migrant counterparts. Multiplicative and additive effects are presented in Table S14. For the majority of ethnicities, further disaggregation resulted in rate ratios that were consistent with the wider group estimate (Table S15). However, within the Mixed ethnic group, the lower consultation rate was primarily driven by individuals from Mixed White and Black Caribbean or African backgrounds, with no evidence of a difference between migrants and non-migrants of Mixed White and Asian or Other Mixed background.

**Figure 5:**
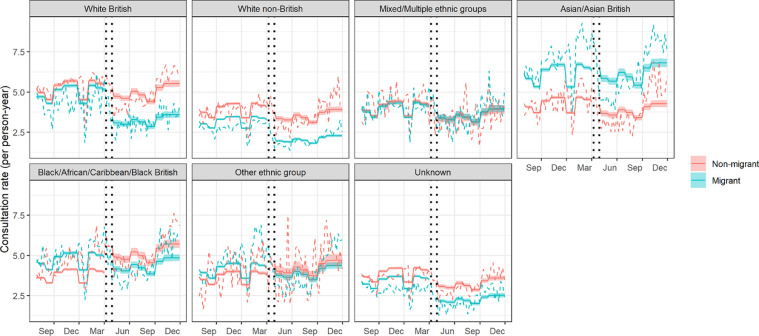
Weekly consultation rates by migration status and ethnicity in England: predicted rates from interrupted time-series analysis (solid line) and actual observed rates (dashed line), truncated view July 2019–November 2020.

Within ethnic groups, the largest reductions in consultation rates during the pandemic in migrants versus non-migrants were in the White British (RR:0.69, 95%CI:0.64–0.73; Figure 5 and Table S16), Black/African/Caribbean/Black British (RR 0.68, 95%CI 0.64–0.73), and White non-British (RR:0.72, 95%CI:0.68–0.77) backgrounds. Within the Black/African/Caribbean/Black British group the largest impact was in individuals of African ethnicity (Figure S2 and Table S17). In the Asian/Asian British ethnic group, the magnitude of the higher consultation rates observed pre-pandemic between migrants and non-migrants further increased during the pandemic (RR:1.11, 95%CI:1.04–1.18; Table S16), which was driven by consultations in the Pakistani and Other Asian groups (Figure S2 and Table S17).

### Sensitivity analyses

In pre-pandemic sensitivity analyses (Figure S3), RRs were numerically lower than the main analysis RRs for both matched cohorts (i.e. [1] matched on age and year when an individual joined a CPRD practice and [2] matched on follow-up). When replacing binary migration status with migration certainty, ‘definite’ and ‘probable’ migrants only differed slightly (Figure S4).

Pre-pandemic estimates obtained from multivariable modelling largely corroborated findings from the ITS analysis over the same period, except in the sensitivity analysis where ‘definite’ migrants had higher rates. This is likely attributed to the lack of age adjustment in the ITS analysis due to using aggregate data. The pandemic amplified the effect of migration on consultations for ‘probable’ migrants (RR:0.87, 95%CI:0.82–0.92), while the effect on ‘definite’ migrants (RR:0.96, 95%CI:0.91–1.02) was not significant (Figure S5 and Table S18).

Results from the ITS cohort sensitivity analyses were generally consistent with those of the main ITS analysis (Figures S6–S8 and Tables S19–S21). In the sensitivity analysis where IMD was removed from the matching process (Figure S7 and Table S20) we observed lower consultation rates in migrants both before and during the pandemic, and found a widening of existing gaps during the pandemic for telephone consultations. In the sensitivity analysis that removed both IMD and practice region from the matching, we also found lower consultations rates for migrants versus non-migrants before the pandemic (Figure S8 and Table S21). However, we only observed lower rates during the pandemic and a greater widening for telephone consultations.

## Discussion

We present findings concerning migrants’ primary care utilisation in England using one of the most comprehensive sources of primary care research data available in the UK.^21^ We show that migrants had lower consultation rates than non-migrants before the COVID-19 pandemic and that the pandemic exacerbated this gap. Our findings also highlight that the effect of being a migrant on primary care utilisation varied significantly by age group and ethnicity.

Lower pre-pandemic consultation rates for migrants in this study are consistent with qualitative and UK-based survey studies of the multiple barriers that migrants face in accessing healthcare.^3^, ^4^, ^5^, ^6^, ^7^ Similarly, lower pre-pandemic consultation rates in migrants have also been reported internationally.^27^, ^28^, ^29^, ^30^ Migrants in young and middle adulthood attended fewer consultations than non-migrants, while the opposite was seen in children and older adults. Higher rates in older adults aligns with previous research^12^ and could be explained by the diminishing ‘healthy migrant effect’ over time^31^ and increasing primary care consultation rates with time post-migration.^11^

The exacerbation of the gap in consultation rates between migrants and non-migrants during the pandemic was more prominent for telephone consultations than face-to-face consultations. It was also more pronounced for children, individuals of White British, White non-British and Black/African/Caribbean/Black British ethnicities, and individuals whose first language was not English. The large impact on migrant children corroborates evidence showing reduced uptake of routine immunisations in England during the pandemic, where barriers to access particularly affected minority ethnic families.^32^^,^^33^ Similarly, disproportionate reductions in primary care during the pandemic have also been reported for migrant children in Ontario, Canada.^34^ Focusing on middle adulthood, evidence prior to this study showed that migrant women experience challenges in accessing maternity care in the UK.^35^ Gender-stratified analysis based on this a-priori hypothesis should be conducted in future in migrants of child-bearing age. The considerable impact on individuals from Black/African/Caribbean/Black British ethnic backgrounds could reflect challenges accessing routine preventive care during the pandemic as a result of health service information not reaching minority ethnic communities.^33^

We found an exacerbation of the gap in consultations rates in individuals whose first language was not English. Whilst we were unable to ascertain English language proficiency using the migration code list and despite incentives for GPs to record main/first language terms between 2008 and 2011 due to the Quality Outcomes Framework,^20^ the recording of a language code in an individual's record could suggest that there may have been communication issues that prompted the staff member to ask about language. Differences between telephone and face-to-face consultations and the impact on individuals whose first language was not English corroborates evidence from England that the shift from in-person to remote primary care exacerbated existing language and access barriers.^18^^,^^36^ In the Asian/Asian British group, further increases during the pandemic to migrants’ already higher pre-pandemic consultation rates build on existing pre-pandemic evidence of higher primary care use in individuals of South Asian ethnicity compared with other ethnic groups.^37^ Possible differences in healthcare needs underlying this finding within Asian/Asian British groups warrant further investigation.

In London, the large gap in telephone consultation rates between migrants and non-migrants that emerged during the pandemic could be due to changes in London's emigration patterns following the onset of the pandemic, which altered its migrant composition.^38^ This change could have seen more migrants experiencing access barriers (e.g. digital and language barriers) remaining in London.

Limitations of our study include the under-recording of migration-related indicators in EHRs, which could result in migrants being misclassified as non-migrants^20^ and a selection bias towards migrants who are more engaged with primary care. Additionally, the way that migration-related information is elicited by GP practices and regions may vary, which could affect whether an individual is classified as a definite or probable migrant and thus, the proportions of definite and probable migrants in a given area. However, this would have a limited effect on the main analysis that examined the entire migrant group. We also observed differences in ethnicity recording between the definite and probable migrant sub-groups; however, as we did not analyse the interaction between ethnicity and migration status in these sub-groups, these differences are unlikely to affect our results. As we reported previously, the migration code list used in this study is less representative of migrants aged over 50.^20^ As a result, findings concerning older migrants should be interpreted with caution. In the context of superdiversity in the UK's migrant population, another limitation is that we did not differentiate between newly arrived and more established migrants who may have very different experiences of accessing healthcare.^39^ Finally, there was a lack of power in the 18-category ethnicity ITS analysis; therefore, these findings should be interpreted with caution.

Our study could also have been affected by changes in size and composition of the migrant population during the study period due to the pandemic itself and/or other factors e.g. the UK's exit from the EU. We found pronounced widening of gaps between migrants’ and non-migrants’ consultation rates in both White British and White non-British groups, who may represent EU migrants. The lack of timely de-registration of migrants who leave GP practices and/or emigrate from the UK could contribute to a greater amount of false follow-up time (i.e. a numerator-denominator bias) and, thus, an underestimation of consultation rates. Finally, we provide quantitative evidence on migrants’ primary care utilisation, which is useful for service planning; however, we did not assess clinical need and, therefore, cannot make firm conclusions regarding the inequity of these differences.

As GP practices continue to use remote consultations,^40^ concerted efforts are needed to ensure all GP services are accessible to migrants. Clinical commissioning groups should address supply-side factors to support efficient and effective use of primary care (e.g. professional interpreting and translation services, culturally responsive service delivery plans) and demand-side factors (e.g. improving migrants’ knowledge of their healthcare entitlements and supporting migrants to make informed decisions about healthcare use during the pandemic and beyond).^18^ Future studies could identify GP practices where there was a lesser widening of the gap between migrants and non-migrants' primary care utilisation during the pandemic, and provide examples of how culturally responsive care and reduction of access barriers have supported migrants’ use of healthcare. Additionally, improvements are needed in both the completeness and accuracy of migration and ethnicity recording in primary care.^41^ Disaggregation of health outcomes by migrant sub-group is needed to better understand the needs of this diverse population^42^ and inform service planning. However, socially excluded migrant sub-groups (e.g. asylum seekers, undocumented migrants, survivors of human trafficking) experience greater barriers accessing NHS services, fear data sharing for immigration enforcement purposes, and are thus rarely captured in routine health data.^43^ Further research is also needed to explore factors that affected migrants and non-migrants differently during the pandemic, whether differences during wave one persisted or reduced to pre-pandemic levels in subsequent waves, and whether specific groups of migrants are adversely affected in the long term.

To conclude, we found that the pandemic impacted migrants’ primary care usage more than that of non-migrants. Although our findings do not provide explanations for this disproportionate impact, they reinforce the role of health services in mitigating service-delivery-related barriers to ensure migrants receive primary care proportionate to their need. Addressing the widening of inequalities will mitigate the future risk of a significantly higher burden of longer term conditions for our healthcare system. This requires policy-makers, commissioners, and service planners to ensure adequate resourcing of primary care to meet the diverse needs of their local migrant and ethnic communities across age groups. Further research is also needed to investigate whether changes in migrants’ primary care usage during the pandemic resulted in inequities in health outcomes.

## Contributors

Conceptualization: RWA, NP, CXZ, RM, SVK, RB, IC-M. Methodology: CXZ, YB, NP, RWA, RM, SVK, PP, AM and AH. Validation: CXZ and YB. Formal Analysis: YB and CXZ. Data curation: CXZ and YB. Writing—original draft preparation: CXZ and YB. Writing—review and editing: YB, CXZ, NP, RWA, RM, SVK, DL, RB, IC-M, PP, GH, AM, AH, and VN. Visualization: YB and CXZ. Supervision: RWA, RM, SVK, and NP. Project administration: CXZ and YB. Funding acquisition: RWA.

CXZ and YB had full access to and verified the underlying study data. All authors have read and agreed to the published version of the manuscript.

## Data sharing statement

This study used pseudonymised patient-level data from CPRD GOLD, which we are unable to publish to protect patient confidentiality. Other researchers can apply to use patient-level data in CPRD GOLD through CPRD's Research Data Governance Process (RDG; https://www.cprd.com/Data–access). All code used to generate these analyses is publicly available https://doi.org/10.5281/zenodo.6345286.

## Declaration of interests

CXZ, YB, and IC-M are employed by the Office for Health Disparities and Improvement in the Department of Health and Social Care and contribute to the development of national guidance and policy in migrant health. CXZ is a Trustee for the international charity Art Refuge. YB's spouse is employed by Elsevier as a Software Engineer. NP received funding from a Clinical Research Training Fellowship from the Wellcome Trust [211162]. RM has received consulting fees from Amgen. SVK acknowledges funding from a NRS Senior Clinical Fellowship (SCAF/15/02), the Medical Research Council (MC_UU_00022/2), and the Scottish Government Chief Scientist Office (SPHSU17). This work was supported by a Wellcome Clinical Research Career Development Fellowship (206602) to RWA and the research costs for the study have been supported by the MRC Grant Ref: MC_PC 19070 awarded to UCL on 30 March 2020 and MRC Grant Ref: MR/V028375/1 awarded on 17 August 2020. RWA received consultancy fees from Doctors of the World, outside the submitted work to complete the 2021 Médecins du Monde Observatory Report. The views expressed are those of the authors and not necessarily those of the Wellcome Trust, UCL, London School of Hygiene and Tropical Medicine, Department of Health and Social Care, Guy's & St Thomas’ NHS Foundation Trust, University of Oxford, University of Glasgow, or the UK Health Security Agency.

## Appendix Supplementary materials

Image, application 1
